# Development of plasma ghrelin level as a novel marker for gastric mucosal atrophy after *Helicobacter pylori* eradication

**DOI:** 10.1080/07853890.2022.2053569

**Published:** 2022-04-13

**Authors:** Hang Yang, Bing Hu

**Affiliations:** Department of Gastroenterology, West China Hospital, Sichuan University, Chengdu, Sichuan, China

To the editor,

The recently published prospective observational research conducted by Hideki Mori et al. revealed that plasma levels of ghrelin correlate well with the level of gastric mucosal atrophy [1]. We read this article with great interest and believe that some issues require further attention.

Ghrelin is secreted by P/D1-cells, which are present from the neck to the base of the oxyntic gland and adjacent to parietal cells (H+) and chief cells (pepsinogen). The ghrelin secretion is physiologically regulated by fasting/feeding, meal composition, chemosensory signalling pathways, body mass index (BMI), insulin and sympathetic/vagus nerves [2].

An inflammation caused by the *Helicobacter pylori* infection can regress within a few months after eradication, and the effects of the inflammation environment (stimulation/inhibition) on mucosal endocrine cells subsequently weaken or disappear. No significant difference was found in ghrelin levels before and after eradication, indicating that the mucosal inflammation environment has little influence on ghrelin secretion compared with gastrin, H + and pepsinogen. The significantly lower level of ghrelin in patients with open-type atrophic gastritis suggests that ghrelin reduction is positively correlated with the loss of oxyntic glands under physiological conditions as no significant changes were found in BMI, adiponectin, glucose and lipid metabolism in this study, which means that the decreased ghrelin is still sufficient for performing its physiological function. Additionally, this observation suggests that ghrelin levels cannot reflect the mucosal atrophy of gastric antrum. Furthermore, the loss of oxyntic glands can be different and similar when considering the same and different ranges of open-type atrophic gastritis. Therefore, ghrelin determination may not reflect the atrophy range. Additionally, if the decreased ghrelin due to cell loss cannot meet the physiological requirements, its secretion will be compensated and increase *via* the feedback mechanism of endocrine hormones. Determining the severity of gastric mucosal atrophy that will cause the decompensation of ghrelin is difficult, to truly reflect the relationship between the ghrelin reduction and the loss of oxyntic glands.
